# Characterisation of the *ex vivo* virulence of *Leishmania infantum* isolates from *Phlebotomus perniciosus* from an outbreak of human leishmaniosis in Madrid, Spain

**DOI:** 10.1186/s13071-014-0499-1

**Published:** 2014-11-07

**Authors:** Gustavo Domínguez-Bernal, Maribel Jiménez, Ricardo Molina, Lara Ordóñez-Gutiérrez, Abel Martínez-Rodrigo, Alicia Mas, Maria Teresa Cutuli, Javier Carrión

**Affiliations:** Department of Animal Health, Faculty of Veterinary Science, Complutense University of Madrid, 28040 Madrid, Spain; Medical Entomology Unit, Department of Parasitology, National Centre of Microbiology, Carlos III Institute of Health, Majadahonda, 28220 Madrid Spain; “Severo Ochoa” Molecular Biology Centre CSIC-UAM, 28049 Madrid, Spain

**Keywords:** *Leishmania infantum*, *Phlebotomus perniciosus*, Leishmaniosis, Outbreak, Virulence, BosqueSur, Madrid, Spain

## Abstract

**Background:**

Since mid 2009, an outbreak of human leishmaniosis in Madrid, Spain, has involved more than 560 clinical cases. Many of the cases occurred in people who live in areas around a newly constructed green park (BosqueSur). This periurban park provides a suitable habitat for sand flies (the vectors of *Leishmania infantum*). Indeed, studies of blood meals from sand flies captured in the area showed a strong association between the insect vector, hares or rabbits, and humans in the area. Interestingly, up to 70% of cases have been found in immunocompetent patients (aged between 46-60 years). This study was designed to evaluate the *ex vivo* virulence of the *L. infantum* isolates from *Phlebotomus perniciosus* captured in this area of Madrid.

**Methods:**

Murine macrophages and dendritic cells were infected *ex vivo* with *L. infantum* strain BCN150, isolate BOS1FL1, or isolate POL2FL7. At different times after infection, the infection indices, cytokine production (IL-12p40 and IL-10), NO release and arginase activities were evaluated.

**Results:**

Using an *ex vivo* model of infection in murine bone marrow-derived cells, we found that infection with isolates BOS1FL1 and POL2FL7 undermined host immune defence mechanisms in multiple ways. The main factors identified were changes in both the balance of iNOS versus arginase activities and the equilibrium between the production of IL-12 and IL-10. Infection with isolates BOS1FL1 and POL2FL7 also resulted in higher infection rates compared to the BCN150 strain. Infection index values at 24 h were as follows: BCN150-infected cells, 110 for infected MØ and 115 for infected DC; BOS1FL1-infected cells, 300 for infected MØ and 247 for infected DC; and POL2FL7-infected cells, 275 for infected MØ and 292 for infected DC.

**Conclusions:**

Our data indicate that *L. infantum* isolates captured from this endemic area exhibited high virulence in terms of infection index, cytokine production and enzymatic activities involved in the pathogenesis of visceral leishmaniosis. Altogether, these data provide a starting point for the study of the virulence behaviour of parasites (BOS1FL1 and POL2FL7) isolated from *P. perniciosus* during the outbreak of human leishmaniosis in Madrid, Spain, and their involvement in infecting immunocompetent hosts.

## Background

*Leishmania infantum* is the etiologic agent of zoonotic visceral leishmaniosis (VL) in humans and other mammals (dogs are the main domestic reservoir hosts) in all countries of the Mediterranean basin [1,2]. VL is a potentially fatal disease in dogs. In several areas of Spain where canine leishmaniosis is highly endemic, seroprevalence of up to 34% has been found, and is considered to be a major problem [3,4]. Other animals including rabbits, hares and cats have also been considered as possible alternative reservoirs of VL [5-7]. Parasites are transmitted from vertebrate animals to humans by the bite of infected female phlebotomine sandflies. No effective vaccine exists against any form of human leishmaniosis. The spectrum and efficacy of current antileishmanial drugs are also limited [8]. All these aspects have recently been reviewed in detail elsewhere [9].

Active VL, mainly diagnosed in young children and in immunocompromised adults, results from uncontrolled multiplication of the parasite in phagocytes of the reticuloendothelial system, and infection is lethal if left untreated. Although the incidence of active disease in humans is relatively low (a range of approximately 5–200 VL cases/year reported by each country), cases are increasing and spreading [10,11].

The environment is undergoing rapid changes because of human actions [12] that also contribute to the changing landscape of leishmaniosis. The latter includes increasing risk factors and new scenarios associated with the zoonotic VL [1,11]. In Spain, a district of abandoned farmland and tailings areas surrounded by a large urban population in the southwest of Madrid was chosen as a site for a newly constructed periurban green park (BosqueSur). This area has been associated with an outbreak of human leishmaniosis, and more than 560 human cases have been detected from July 2009 to date; notably, 70% of the cases have been found in immunocompetent patients aged between 46-60 years [13,14].

Current studies suggest that this outbreak was due to human-induced environmental changes (land cover and land use) that created regional combinations of eco-epidemiological conditions; these conditions may influence patterns of sand fly vector (*P. perniciosus*) distribution and may result in infections of unusual mammalian hosts of *L. infantum* [14-17]. In this context, xenodiagnostic studies performed with wild lagomorphs (hares and rabbits) captured in the green park demonstrate that these animals play some role as unusual reservoirs that transmit *L. infantum* parasites to *P. perniciosus*. Moreover, studies of blood meal preferences of *P. perniciosus* caught in the area revealed that the insect vector prefers to feed on rabbits and hares, but will occasionally feed on other hosts, such as dogs or humans [14,18,19]. Altogether, published data support the idea that the peridomestic and sylvatic transmission cycles overlap in the area, and that infected dogs are not essential to maintaining the transmission cycle of *L. infantum*.

Since this outbreak started, lagomorphs have attracted the interest of researchers in Spain. Chicharro *et al.* [20] suggested that the outbreak was not caused by a ‘new’ emerging genotype. Molecular typing studies show that the isolates involved in the outbreak belonged to the ITS LOMBARDI subtype of *L. infantum*, as did those isolated in different parts of Madrid since at least 1992.

However, the question regarding how parasites have been able to infect a high number of immunocompetent patients during the current outbreak remains important and unanswered. The aim of the present study was to evaluate the *ex vivo* virulence of the *L. infantum* isolates recovered from *P. perniciosus* that were captured in the area of human leishmaniosis in Madrid, in comparison with that of another well-characterized strain.

## Methods

### Mice and parasites

Six-week-old female BALB/c mice were purchased from Harlan Interfauna Ibérica (Barcelona, Spain). The animal research described in this manuscript complied with Spanish (Ley 6/2013) and European Union legislation (2010/63/UE). The protocols used were approved by the Animal Care Committee of Complutense University of Madrid.

*L. infantum* parasites were used in this study. Two isolates named IPER/ES/2012/BOS1FL1 (BOS1FL1) and IPER/ES/2012/POL2FL7 (POL2FL7) were isolated from *P. perniciosus* captured in the focus of Madrid using CDC light traps. BCN150 is a well characterized strain (M/CAN/ES/96/BCN150 zymodeme MON-1) that was isolated from a dog with active VL [21]. This strain has traditionally been used in our laboratory’s experiments related to VL [22-24] and by others in canine leishmaniosis studies [21,25,26]. All *L. infantum* parasites were previously passed through golden hamsters (*Mesocricetus auratus*). Two months after infection, infected spleen samples were cultivated in NNN medium at 26-27°C for 4-7 days, until promastigotes expanded. These were then used for *ex vivo* infection experiments in this study.

### Differentiation of bone marrow-derived cells

Macrophages (BMMø) and dendritic cells (BMDC) were differentiated *in vitro* from bone marrow stem cell progenitors. Briefly, BALB/c mice were euthanised, and cell suspensions were obtained by flushing the femurs and tibias with Hank’s Balanced Salt Solution (HBSS). Cells were collected by centrifugation and cultured in DMEM containing 2 mM L-glutamine, 0.1 mM nonessential amino acids, 10 mM HEPES, antibiotics, and 10% heat-inactivated foetal bovine serum (FCS). BMMø were cultured in non-tissue culture treated Petri dishes for 8 days in the presence of M-CSF (Peprotech) at a concentration of 50 ng/ml. At days 3 and 6, half of the supernatants were discarded and replaced with 5 ml of fresh medium containing M-CSF (50 ng/ml). BMDC were cultured for 10 days in 25 ml flasks in the presence of GM-CSF (20 ng/ml, Peprotech). This supplemented DC medium was added at days 0, 3 and 6. After their respective periods of differentiation, BMMø and BMDC were obtained as previously described [27,28] and displayed a phenotype highly enriched in F4/80^+^ or CD11c^+^ cells (~95%), respectively.

### Infection index of bone marrow-derived cells

BMMø and BMDC were cultured overnight in the presence or absence of IFN-γ (500 ng/ml) into LabTek culture chamber slides (Thermo Scientific) using 5 × 10^4^ cells per chamber. On the following day, parasites were added to cells at a ratio of 10:1 parasites: BMMø or BMDC. After 4 h of incubation at 37°C, a time point that reflects initial infection, extracellular parasites were removed by washing, and cells were incubated in fresh medium for 24 h, 48 h and 72 h. After Giemsa staining, cells were mounted with Coverquick, and 400 cells were counted in duplicate in a microscope Olympus BX41. The percentage of infected cells and the mean of the number of amastigotes per infected cell were evaluated. The infection index was calculated by multiplication of both parameters to account for the overall parasite load, as previously described [29].

### Cytokine production, nitric oxide (NO) release and arginase activity

BMMø and BMDC (1 × 10^6^ cells/ml) were cultured overnight into 24 well non-tissue culture treated plates. Thereafter, parasites were added (at a ratio of 10:1, as above) to cells and extracellular promastigotes were removed by washing after 4 h. Subsequently, cells were stimulated or not with LPS (1 μg/ml). In some cases, culture supernatants were collected after 24 h for cytokine quantification (IL-12p40 and IL-10, BD Pharmigen) by ELISA according to the manufacturer’s instructions. At 96 h, supernatants from the other wells were collected and nitric oxide NO release was measured as nitrite concentration using Griess reagent. Then, BMMø and BMDC were then harvested and used to determine of arginase activity as previously described [30].

### Statistical analysis

Statistical analyses were performed using SigmaPlot version 11.0 (Systat Software, Inc). Significant differences between different strains were determined and are designated with asterisks as follows: *P <0.05, **P <0.01, ***P <0.001.

## Results and discussion

In this study, we defined virulence in terms of measuring the abilities of a well-characterised *L. infantum* strain and two isolates from *P. perniciosus* to induce factors contributing to disease under controlled conditions. In this *ex vivo* context, we quantified virulence, using assessment of both infection index and the quality of immune responses due to infection, as shown below.

### Evaluation of infection index and related markers

**Figure 1:**
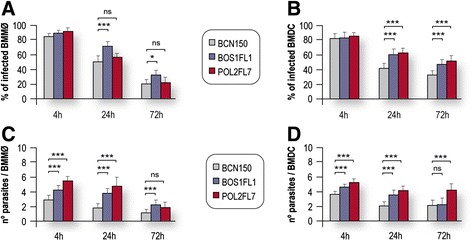
***Ex vivo*** **differential virulence of** ***L. infantum*** **parasites.** The time course of infection was followed counting **(A, B)** infected cells and **(C, D)** the number of parasites per infected cell. In cases in which the data was not normally distributed (P <0.05), a 2-sample t-test could not be used. We used the Mann-Whitney U test instead. Each value represents the mean ± SD from three independent experiments. Significant differences between BCN150 strain and the other isolates were determined, as indicated. *P <0.05, **P <0.01, **P <0.001; ns, not significant.

**Figure 2:**
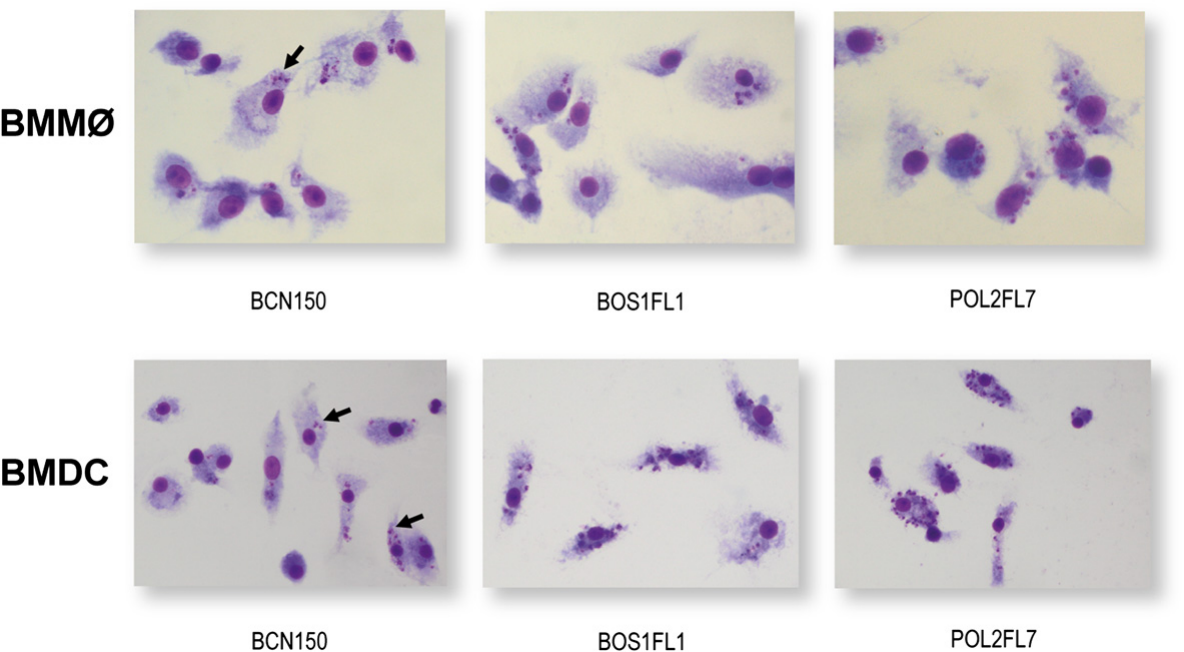
**Examination of infection of BMMø and BMDC at 24 h after infection.** Infected cultures were washed to remove free parasites, fixed in methanol, stained with Giemsa and examined under a light microscope. Intracellular amastigotes (arrows) were clearly visible.

We infected murine BMMø and BMDC and followed the progression of infection for 72 h. At the initial time point studied (4 h), more than 80% of the cells were infected, and there were no significant differences between parasite isolates in the total percentage of infected BMMø and BMDC (Figure 1A-B). However, significant differences were observed in BMDC at 24 h and 72 h after infection (see Figure 1B). Nevertheless, the analysis of parasite load per infected cell showed that both BOS1FL1 and POL2FL7 isolates were more effective in the invasion of BMMø and BMDC compared to the BCN150 strain. In this study, the median values of parasite load per BCN150-infected cells were considered representative samples (reference values) of our experiments using this well-characterized *L. infantum* strain. As expected at 24 h after infection [29,31], we detected a reduction in BCN150-infected the intracellular parasite load (for all three strains) compared to cells at the initial time point of infection (Figure 1C-D), which was more apparent in BMMø and BMDC. As previously described [31], the number of BMDC containing intracellular parasites dropped significantly between 4 h to 24 h. After the slight decrease observed at 24 h, the mean number of amastigotes per infected BMDC decreased at later time points, although the parasite burdens in cells infected with the BOS1FL1 and POL2FL7 isolates remained greater compared to cells infected with the BCN150 strain. Therefore, both the BOS1FL1 and POL2FL7 isolates were more resistant to BMMø and BMDC leishmanicidal activities, as displayed by significantly higher values of infection indices at all time points studied, compared to those observed in BCN150 infection (Table 1). Thus, differences in amastigotes content were clearly visible under a light microscope after 24 h of infection (Figure 2). Given these results, BOS1FL1 and POL2FL7 isolates may be considered to be more infective than the BCN150 strain.

**Table 1:** **Infection indeces**

**Infection index**	**BMMØ**			**BMDC**		
	**BCN150**	**BOS1FL1**	**POL2FL7**	**BCN150**	**BOS1FL1**	**POL2FL7**
**4 h after infection**	252 ± 50	396 ± 50*	552 ± 50*	319 ± 25	399 ± 30*	475 ± 50*
**24 h after infection**	110 ± 50	300 ± 50*	275 ± 50*	115 ± 25	247 ± 20*	312 ± 30*
**72 h after infection**	30 ± 10	60 ± 15*	40 ± 10*	102 ± 10	137 ± 15*	292 ± 20*

In order to determine the overall parasite burdens, an infection index value was calculated for each time after infection by multiplying the mean percentage of infected cells by the mean number of amastigotes per infected cell. The resulting values are expressed in arbitrary units. One representative experiment out of three is shown. The mean ± SD are shown. *P <0.05.

### Characterisation of the quality of immune response due to *ex vivo* infection

Infection with *L. infantum* is known to lead to cytokine secretion by host cells that will dictate the nature of T-cell responses. The quality of these immune responses is influenced by the equilibrium between the production of pro-inflammatory cytokines (mainly represented by IL-12) and anti-inflammatory cytokines (mainly represented by IL-10). In VL, IL-10 suppresses IFN-γ and IL-12 production, while disease regression is associated with optimal IL-12p40 production by DC and Mø that drives a predominant T helper 1 response. Th1 T cell responses result in IFN-γ production, type 1 antibody responses, classical macrophage activation and subsequent NO-mediated killing effector functions [32,33]. These activities are essential to destroy the invading pathogens [34,35].

**Figure 3:**
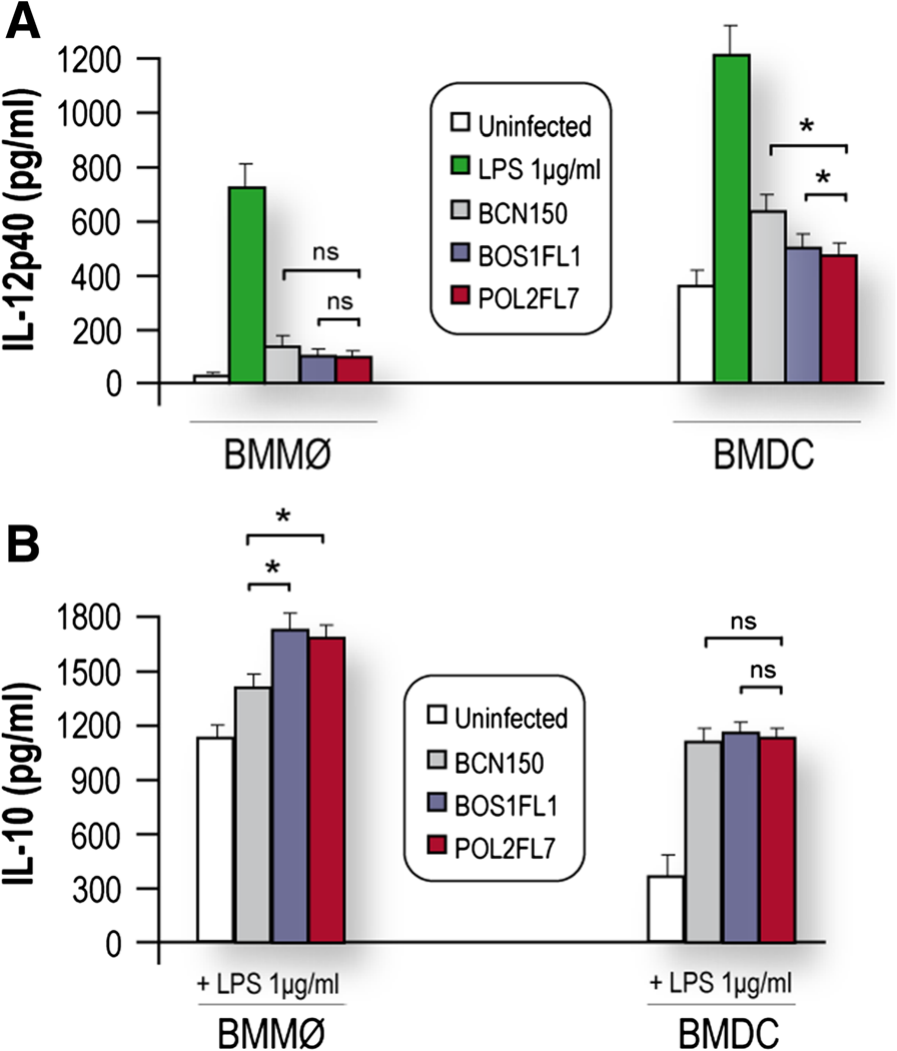
**Effect of infection on BMMø and BMDC cytokine production.** After 4 h of infection with *L. infantum*, cells were cultured in the presence or absence of LPS (1 μg/ml) and levels of **(A)** IL-12p40 and **(B)** IL-10 in 24 h culture supernatants were quantified by ELISA. One representative experiment of three is shown. The mean ± SD are shown. *P <0.05 indicates significant differences between BCN150 and the other isolates; ns, not significant.

Thus, controlled parallel experiments were performed in the *ex vivo* murine model of infection to assess the ability of parasites (BOS1FL1, POL2FL7 and BCN150) to modulate cytokine synthesis in LPS- activated BMMø and BMDC. *Leishmania* infection induced lower amounts of IL-12p40 production compared to amounts observed when cells were stimulated with only LPS (Figure 3A), which is consistent with previous research [36]. Interestingly, the production of IL-12p40 was decreased mainly in BOS1FL1- and POL2FL7-infected BMMø and BMDC (*P* <0.05), compared to amounts observed during infection with the BCN150 strain (Figure 3A). BMMø infected with BOS1FL1 and POL2FL7 isolates produced higher levels of IL-10 (*P* <0.05) compared to those produced in cultures of cells infected with the BCN150 strain. In contrast, no significant differences were found for the IL-10 levels in BMDC after 24 h of infection with any of three *L. infantum* strains (Figure 3B). Altogether, these data show that BOS1FL1- and POL2FL7-infected BMMø produced higher levels of the anti-inflammatory cytokine IL-10, while these isolates interfered significantly in optimal IL-12p40 production by *Leishmania*-infected BMDC. These results suggest that there are two ways by which these isolates may alter the defences of host cells, depending on whether the infection involves BMMø or BMDC.

**Figure 4:**
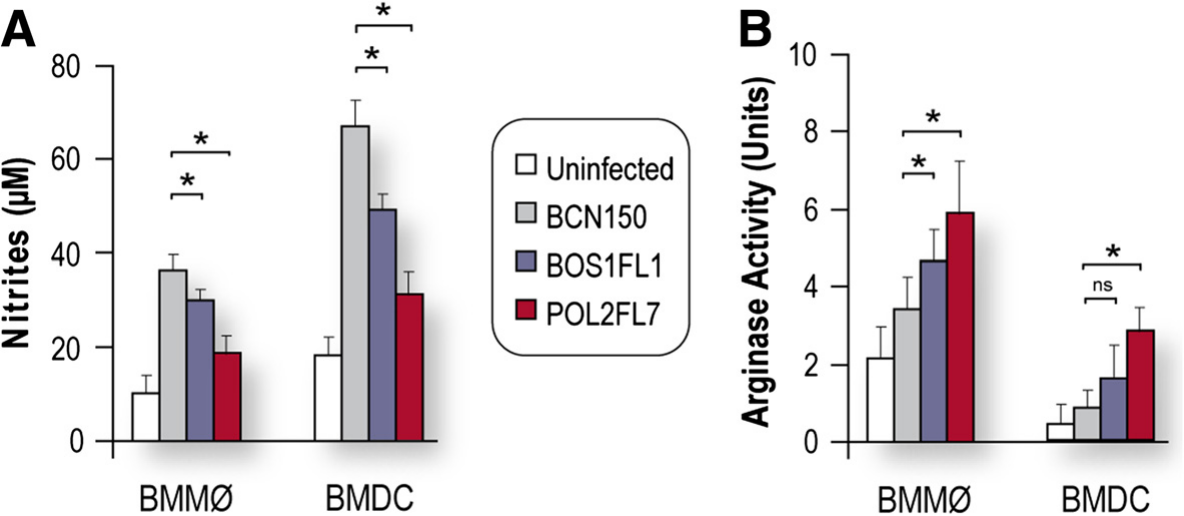
**NO release and arginase activity at 96 h after infection. (A)** Immediately after 4 h of infection, cells were stimulated with LPS (1 μg/ml) and nitrite levels in culture supernatants harvested 96 hours later were determined using Griess assay method. **(B)** Non-stimulated cells were lysed and the total arginase activity was determined. Data are presented as mean ± SD and are representative of three different experiments with similar results. *P <0.05 indicates significant differences between BCN150 and the other isolates; ns, not significant.

To investigate whether BOS1FL1, POL2FL7 or BCN150 parasites can affect host cell responses that kill intracellular parasites, we compared the levels of NO produced and arginase activity in *ex vivo*-infected BMMø and BMDC at 96 h after infection. We found significantly reduced NO production (Figure 4A) in supernatants from BOS1FL1- and POL2FL7-infected BMMø and BMDC in the presence of LPS compared to those obtained in BCN150-infected cells. The results also showed that *ex vivo* infections lead to augmented arginase activity in infected cells, and confirmed previous investigations using different *Leishmania* species [37-39]. In agreement with data above, arginase activity was increased in BMMø and BMDC infected with BOS1FL1 and POL2FL7 (Figure 4B), compared to levels of activity in cells infected with BCN150. It is well known that arginase, an enzyme induced by Th2 cytokines, is a hallmark of host cells and is responsible not only for the transformation of L-arginine into polyamines, contributing to the growth of *Leishmania* parasites, but arginase is also important for parasite infectivity [40]. This increased arginase activity in phagocytic cells is believed to deplete L-arginine availability and to competitively inhibit iNOS activity and NO production [37,41,42]. Data in the present study clearly show that the increased arginase activity in BMMø and BMDC infected with BOS1FL1 and POL2FL7 is likely to overcome the parallel NO release, resulting in reduced leishmanicidal activity in host cells.

## Conclusions

This study was designed to examine the *ex vivo* virulence of *L. infantum* strains isolated from an area associated with human leishmaniosis in Madrid and to assess the potential implications in this human leishmaniosis outbreak. Our data indicate that *L. infantum* isolates from an endemic area exhibited high virulence in terms of infection index, cytokine production and enzymatic activities involved in the pathogenesis of VL. These data provide a starting point for the study of the virulence behaviour of parasite isolates (BOS1FL1 and POL2FL7) from an outbreak of human leishmaniosis in Madrid (Spain), including their involvement in infecting immunocompetent hosts. Further studies will be needed to determine whether these findings are observed in an *in vivo* immunocompetent murine model of VL.
